# The role of phosphate-containing medications and low dietary phosphorus-protein ratio in reducing intestinal phosphorus load in patients with chronic kidney disease

**DOI:** 10.1038/s41387-019-0080-2

**Published:** 2019-04-03

**Authors:** Jiameng Li, Liya Wang, Mei Han, Yuqin Xiong, Ruoxi Liao, Yupei Li, Si Sun, Anil Maharjan, Baihai Su

**Affiliations:** Department of Nephrology, West China Hospital, Sichuan University, 610041 Chengdu, China

## Abstract

Chronic kidney disease-mineral and bone disorder (CKD-MBD) is a common complication in patients experiencing end-stage renal disease (ESRD). It includes abnormalities in bone and mineral metabolism and vascular calcification. Hyperphosphatemia is a major risk factor leading to morbidity and mortality in patients with chronic kidney disease. Increased mortality has been observed in patients with ESRD, with serum phosphorus levels of >5.5 mg/dL. Therefore, control of hyperphosphatemia is a major therapeutic goal in the prevention and treatment of CKD-MBD. The treatment of hyperphosphatemia includes decreasing intestinal phosphorus load and increasing renal phosphorus removal. Decreasing the intestinal load of phosphorus plays a major role in the prevention and treatment of CKD-MBD. Among the dietary sources of phosphorus, some of the commonly prescribed medications have also been reported to contain phosphorus. However, drugs are often ignored even though they act as a potential source of phosphorus. Similarly, although proteins are the major source of dietary phosphorus, reducing protein intake can increase mortality in patients with CKD. Recently, the importance of phosphorus/protein ratio in food have been reported to be a sensitive marker for controlling dietary intake of phosphorus. This review summarizes the progress in the research on phosphate content in drugs as an excipient and the various aspects of dietary management of hyperphosphatemia in patients with CKD, with special emphasis on dietary restriction of phosphorus with low dietary phosphate/protein ratio.

## Introduction

Chronic kidney disease-mineral and bone disorder (CKD-MBD) is a recently acknowledged metabolic abnormality, proposed in 2006 by the Kidney Disease: Improving Global Outcomes (KDIGO) working group. This distinct naming of varied clinical syndromes (CKD-MBD) that develop due to impaired mineral regulation in chronic kidney disease (CKD) was to differentiate it from the histologically well-defined renal osteodystrophy^1^. CKD-MBD is considered to be a progressive disorder, affecting the homeostasis among the renal, skeletal, and cardiovascular (CV) systems, thereby leading to a kidney–bone–vascular axis pathophysiological hypothesis^1,2^. The classical clinical manifestations of CKD-MBD defined by the KDIGO clinical practice guidelines also reaffirms the kidney–bone–vascular axis hypothesis. The clinical features of CKD-MBD are primarily due to the compromised renal excretion of minerals, leading to abnormality in circulating calcium, phosphorous, parathyroid hormone (PTH), and vitamin D. The secondary effects of this renal insufficiency include abnormalities in bone turnover, mineralization, volume, linear growth, strength, and ectopic calcification (vascular and soft tissues)^3^.

Among the different pathogenic factors, phosphate homeostasis is one of the initiating factors for the onset of CKD-MBD, which is initiated during early stages of CKD. This anomaly in phosphate homeostasis was evidently shown by elevated fibroblast growth factor 23 (FGF23) levels (hormone-regulating phosphate excretion) in early stages of CKD^4^. The two main homeostatic mechanisms that regulate circulating phosphates are intestinal absorption and renal excretion. While intestinal absorption depends on the dietary pattern, renal excretion promotes removal of excess phosphates even in case of phosphate-rich diet through regulation of FGF23 and PTH^5,6^. In individuals with normal renal function, this is facilitated by decreased reabsorption of phosphates, which is mediated by downregulation of sodium phosphate cotransporters in the proximal tubule of the nephrons^7,8^. However, in case of patients with CKD, the progressive reduction in glomerular filtration leads to further decrease in phosphate reabsorption, which compensates the renal insufficiency during the early stages of CKD. In CKD stages 3 to 5 (eGFR < 60 mL/min/1.73 m^2^), the phosphate homeostatic mechanism is totally lost leading to increase in circulating phosphates and inorganic phosphorous (Pi), which is defined as hyperphosphatemia^9,10^.

Although the pathogenic basis of hyperphosphatemia in CKD is renal insufficiency, the clinical manifestations of CKD-MBD are mainly due to the adaptive response mediated by PTH and FGF23. Elevated PTH induces excess bone resorption of phosphates, which exceeds bone formation, leading to osteoporosis and other skeletal structure abnormalities. Furthermore, since skeletal structures could no longer act as phosphate reservoirs, soft tissues and vasculatures become secondary reservoirs, thereby leading to their calcification and increasing CV morbidity and mortality in patients with CKD^11–14^.

Hyperphosphatemia in patients with CKD, apart from inducing secondary hyperparathyroidism and renal osteodystrophy, CV calcification is also an important prognostic factor for morbidity and mortality in patients with end-stage renal disease (ESRD) undergoing dialysis^15–20^. The prognostic role of serum phosphorous as a modifiable risk factor has also been reported in multiple observational studies, highlighting the potential effect of lowering blood phosphorous (Pi and phosphates) in CKD-MBD^17,21,22^. This was also substantiated in a meta-analysis including a total of 4651 patients with non-dialysis CKD, in whom a 35% increase in mortality was observed per mg increase in phosphate^23^.

The therapeutic management of hyperphosphatemia includes decreasing intestinal absorption of phosphorus and increasing its renal removal. The former includes dietary restriction to reduce phosphorus intake^24–27^ and a phosphorus binder to reduce oral phosphorus absorption^15,16,28^. The latter includes regular dialysis to remove blood phosphorus, which is considered to be of limited value in removal of phosphates, highlighting the importance of dietary restriction^29,30^. Owing to the fact that proteins are the main source of dietary phosphates, which is difficult to restrict, dietary control of phosphates has its own pitfalls.^10^ Furthermore, when it comes to patients with CKD, an often ignored source of dietary phosphate is the long-term multiple medications, some of which have been reported to contain phosphate^31,32^. This review provides an overview of the various aspects of dietary management of hyperphosphatemia in patients with CKD, with special emphasis on reducing intestinal phosphorus load in those patients by careful prescription of phosphate-containing medications and dietary restriction in terms of low dietary phosphate/protein ratio. To the best of our knowledge, this is the first review highlighting the phosphorus content in medications prescribed to patients with CKD.

## Phosphorus content in CKD medication

Other than dairy or protein-based food products, which are rich in dietary phosphates, there is yet another source of dietary phosphorus that has remained unrecognized, i.e., the phosphorus content of medications prescribed for patients undergoing dialysis^32,33^. In pharmaceutical preparation, phosphates are used both as an active pharmaceutical ingredient (API; such as bisphosphonates) or as a drug counterion (e.g., betamethasone sodium phosphate), or most commonly as an excipient (e.g., anhydrous calcium hydrogen phosphate). This also includes some of the available drugs used for the treatment of CKD in which the most common role of phosphorus is as an excipient^34,35^.

### Phosphorus as a pharmaceutical excipient

The largely unrecognized role of phosphorus content in medication is mainly due to its role in pharmaceutical preparations. It is mostly used as a pharmaceutical excipient, which in most cases is reported on the packaging label without the precise concentration^33^. Phosphates as excipients are most commonly used as diluents, buffers to prevent pH fluctuations, and provide the required density for the preparation, thereby facilitating appropriate route of administration^32,36^. Although excipients are often considered as pharmacologically inert ingredients, they could also have iatrogenic effects as shown by diethylene glycol in sulfanilamide, which caused acute kidney injury, killing more than 100 people in the US^37^. The phosphorus-containing excipients in drugs are not inert as they increase the phosphorus intake and thereby increase the blood phosphorus levels that could have potential iatrogenic effects^35^. Although the phosphorus content in excipients usually contribute to only a small fraction of the recommended daily intake, it is still clinically significant in terms of treating patients with CKD because most of these patients will be on multiple medications^38^. Polypharmacy is a major problem for patients with ESRD due to the burden of multiple comorbidities and dialysis-related complications caused by it^38,39^. In a study of 233 patients undergoing chronic hemodialysis (HD), more than a quarter of the patients were prescribed >25 tablets per day with a median daily pill burden of 19 (refs. ^35,38^). If most of the medications consumed by patients undergoing dialysis contain phosphate, then this will significantly contribute to daily phosphorus intake of these patients, which may contribute to the progression of CKD-MBD. Although the number of phosphate-binder pills taken by the patient is not accounted for in this study, the phosphate burden due to medication is significant^35^.

### Phosphorus-containing drugs should not be overlooked

Patients with CKD are usually prescribed a daily dose of multiple medications, and hence, drugs are an important source of phosphorus intake that cannot be ignored. Commonly used drugs for long-term oral administration in patients with CKD alleviate comorbidities related to CV system and central nervous system (CNS). The different medications include antihypertensives, anticoagulants, immunosuppressants, hypoglycemic agents, lipid-lowering drugs, analgesics, antihistamines, antidepressants, anti-gout drugs, and gastrointestinal drugs^35,36^. A potential confounder in estimating the medicinal phosphorus content is the wide variation observed with different formulations of the same drug from different manufactures, which has been highlighted in previous studies^31,35^. Although the label usually mentions that the drug contains phosphorus (usually shown in the excipient list), the label does not specify the exact quantity of phosphorous present^31^.

The extent of the problem was highlighted by a landmark study by Sherman et al., who reviewed the phosphorus content mentioned in the package inserts of hemodialytic medications and also confirmed the precise concentration of phosphorus by colorimetric methods. They analyzed different doses of both the branded and generic forms of multiple drugs and reported dose-independent variations in phosphorus content between branded and generic formulations. They identified 12 prescription drugs containing phosphorus, with 9 of them being prescribed for a daily dose of >10 mg. Phosphorus was identified in both branded and generic forms of amlodipine, lisinopril, paroxetine, and bisoprolol. On the basis of their preliminary study, they also hypothesized that when the branded form of a drug is phosphorus-free, then the generic form may also be phosphorus-free. Although contrary to their hypothesis, they observed generic form of paroxetine to contain lower concentration of phosphorus than the branded version^31^. In a subsequent large-scale analysis by the same authors, 200 most commonly prescribed branded medications used in the dialysis centers of the US-based Dialysis Clinic, Inc. (Nashville, TN) were analyzed and reported 23 branded formulations (11.5%) to contain phosphorus in their label. As they could not ascertain the phosphorus content from the drug manufacturers, they estimated the phosphorus content of multiple doses of branded and generic formulations by spectroscopy-based method. They reported the amount of phosphorus to vary from 1.4 mg/tablet (clonidine: 0.2 mg; BluePoint Laboratories, Dublin, Ireland) to 111.5 mg (paroxetine: 40 mg; GlaxoSmithKline, Philadelphia, PA). The branded versus generic comparison revealed results similar to their earlier study. Furthermore, in order to confirm their hypothesis with respect to the absence of phosphorus in generic form, provided the branded form is phosphorus-free, they analyzed 91 generic drugs and found phosphorus in only 1 generic drug (Pravachol, Eon) despite the absence of phosphorus in its branded form^31,33^. The phosphate content of the various commonly prescribed drugs is represented in Table 1.

**Table 1:** Phosphorus content in commonly prescribed medications to patients undergoing dialysis

Medication: Dosage (mg)	Content of Available Phosphorus (mg)	Manufacturer
Amlodipine		
5	13.4	Pfizer Inc
5	3.8	Camber Pharm
10	7.9	Camber Pharm
10	40.1	Qualitest
5	4.9	Lupin
10	8.6	Lupin
5	14.0	Greenstone Brand
2.5	25.99	Actavis Speciality Pharmaceuticals Co.
5	51.99	Actavis Speciality Pharmaceuticals Co.
10	103.9	Actavis Speciality Pharmaceuticals Co.
2.5	NA	Auro Pharma Inc.
5	36	Auro Pharma Inc.
10	72	Auro Pharma Inc.
2.5	20.9	JAMP Pharma Corporation
5	41.88	JAMP Pharma Corporation
10	83.8	JAMP Pharma Corporation
2.5	22.4	Laboratoire Riva Inc.
5	44.8	Laboratoire Riva Inc.
10	89.6	Laboratoire Riva Inc.
2.5	NA	Mint Pharmaceuticals Inc.
5	6.9	Mint Pharmaceuticals Inc
10	13.8	Mint Pharmaceuticals Inc
2.5	22.1	Pharmascience Inc.
5	44.3	Pharmascience Inc.
10	88.6	Pharmascience Inc.
2.5	NA	Pfizer Canada Inc.
5	44	Pfizer Canada Inc.
10	88	Pfizer Canada Inc.
2.5	29.1	Sandoz Canada Inc.
5	58.3	Sandoz Canada Inc.
10	116.6	Sandoz Canada Inc.
2.5	22.7	Sivem Pharmaceuticals
5	45.4	Sivem Pharmaceuticals
10	90.8	Sivem Pharmaceuticals
2.5	NA	Teva Canada Ltd.
5	82.8	Teva Canada Ltd.
10	165.6	Teva Canada Ltd.
Lisinopril		
5	3.6	Lupin
20	7.4	Lupin
5	18.4	Blue Point Laboratories
10	32.6	Blue Point Laboratories
20	20.8	Blue Point Laboratories
30	27.4	Blue Point Laboratories
40	30.8	Blue Point Laboratories
10	21.4	Merck
20	22.0	Merck
10	28.9	Sandoz
20	30.7	Sandoz
40	26.2	Sandoz
Rosuvastatin		
5	1.9	AstraZeneca
10	1.8	AstraZeneca
20	3.8	AstraZeneca
Paroxetine		
10	17.1	Aurobindo
20	55.8	GlaxoSmithKline
40	111.5	GlaxoSmithKline
10	147.9	Mint Pharmaceuticals
20	295.8	Mint Pharmaceuticals
30	443.7	Mint Pharmaceuticals
Azithromycin: 250	30.7	Pfizer Inc
Glyburide: 5	27.6	Aurobindo
Megace (Megestrol acetate): 40	28.8	Par Pharmaceutical
Repaglinide		
0.5	7.2	Paddock
1	9.4	Caraco
Sitagliptin		
25	7.3	Merck
50	13.2	Merck
Sertraline		
50	4.3	Pfizer Inc
100	8.6	Pfizer Inc
25	2.1	Greenstone Brand LLC
50	4.5	Greenstone Brand LLC
100	8.7	Greenstone Brand LLC
Rabeprazole: 10	2	Eisai China
Bisoprolol		
5	28	Pharmascience Inc.
10	31	Pharmascience Inc.
5	83.6	Sandoz Canada Inc.
10	80.1	Sandoz Canada Inc.
5	83.5	Sivem Pharmaceuticals
10	80.1	Sivem Pharmaceuticals
5	61.9	Teva Canada Ltd.
10	61.9	Teva Canada Ltd
5	28	Pharmascience Inc.
10	31	Pharmascience Inc.
5	24.5	Eon
10	23.8	Eon
5	0	Mylan
10	14.8	Aurobindo
10	24.6	Sandoz
Zopiclone		
3.75	41.4	Teva Canada Ltd.
5	41.4	Teva Canada Ltd.
7.5	41.4	Teva Canada Ltd.
Quetiapine		
25	8	Teva Canada Ltd.
100	34	Teva Canada Ltd.
150	25	Teva Canada Ltd.
Oxycodone		
5	5.21	Pharmascience Inc.
10	4.11	Pharmascience Inc.
20	8.22	Pharmascience Inc.
Acetaminophen		
8 mg Codeine	60	Teva Canada Ltd.
15 mg Codeine	60	Teva Canada Ltd.
30 mg Codeine	60	Teva Canada Ltd.
Clonidine		
0.1	2.2	Boehringer Ingelheim
0.2	1.4	Blue Point Laboratories
0.2	3.5	Unichem

Source: Bibliographic references^31,35^

The phosphorus content of branded versus generic formulation issue was also addressed by a similar study conducted by Shimoishi et al., with 22 widely prescribed medications for patients undergoing HD in Japan. They also reported branded formulations such as amlodipine and paroxetine to be rich in phosphorus content among the study drugs. In case of paroxetine, all the generic formulations contained lower levels of phosphorus compared with the branded formulations, which was in concurrence to the study by Sherman et al., whereas in case of amlodipine, the results were mixed with few generic formulations having higher levels of phosphorus compared with the branded formulations^31,32^. However, as the number of drugs examined by Shimoishi et al. was relatively low, the precise nature of branded and generic formulations may require further studies.

In a similar study, Nelson et al. calculated both the consumed and estimated medicinal phosphorus loads in 101 Canadian patients undergoing HD. They analyzed 1744 drug formulations of 124 different drugs prescribed to patients undergoing HD and found that a total of 185 (11%) drug formulations contained phosphorus as per the drug monographs obtained from the Health Canada Drug Database. In order to find the precise amount of phosphorus, they contacted 26 drug manufacturers, out of which 18 manufacturers revealed the phosphorus content. Among the phosphorus-containing drugs, 65% of the drugs were prescribed for CNS-related comorbidities, followed by 24% for CV anomalies. The percentage of formulations with phosphate was very high among codeine (98%), zopiclone (95%), and quetiapine (73%). In total, 31 patients (30%) were taking at least one phosphate-containing medicine per day. Among them, the median phosphate burden from prescribed medications was reported to be 111 mg/day, which corresponds to approximately 10% of the KDIGO-recommended daily dosage (800–1000 mg)^35,40^.

The quantum of phosphate-containing CKD drugs was also reported by a retrospective database analysis by Sultana et al. They utilized the PubChem and other publicly available databases to confirm the presence of phosphate in the medicinal products used to treat patients with CKD. They investigated a total of 3779 medicines consumed by an Italian cohort of 1989 patients with CKD and found that 266 medicinal products containing phosphorus (7%) were consumed by 1381 patients with CKD (70%) over a median follow-up of 6 years. Further investigations revealed that in majority of those medications phosphate was derived from the excipient (94.4%) rather than the active moiety (0.8%) or the counterion (8.3%)^34^.

One important observation made in all the above studies was the diverse amounts of phosphorus present in different formulations of the same drug, which highlights the difficulty in controlling medicinal phosphorus intake. The proportion of different groups of phosphorus-containing medications from the reported studies is summarized in Table 2.

**Table 2:** Proportion of medications containing phosphate as an excipient

Medications by category	Number of formulations reviewed	Number of formulations containing hosphorus	Phosphorus-containing medications (%)
Calcium channel blockers	39	20	51
ACE inhibitors and angiotensin-receptor blockers	89	9	10
Beta blockers	39	9	23
Cholesterol-lowering therapy	38	8	21
Pain medicines	107	48	44.8
Anxiolytic agents	42	0	0
Antidepressants	173	10	5.7
Antipsychotics	54	19	35.1
Drugs used in diabetes	239	57	23.8
Psycho-analeptics	738	142	19.2
Antihypertensives	927	162	17.5
Drugs for acid-related disorders	274	45	16.4
Thyroid therapy	50	4	8
Lipid-modifying agents	354	28	7.9
Analgesics	163	11	6.7
Immunosuppressants	67	4	6
Antihistamines for systemic use	105	3	2.9
Drugs for chronic obstructive airway diseases	394	9	2.3
Antithrombotic agents	92	2	2.2
Cardiac therapy	238	5	2.1
Anti-gout preparations	12	0	0
Hormones	69	12	17
Vitamins	7	2	29
Gastrointestinal medications	213	2	0.9
Blood coagulation andformation	26	0	0

Source: bibliographic references^35,36^

### Phosphorus content in commonly used oral drugs

In view of the KDIGO guideline recommendations for dietary phosphorus intake in patients with CKD, the intestinal load of phosphorus through oral medications took precedence. Moreover, due to the practical difficulty involved with other routes of administration in patients with CKD, a bulk of the medicines were administered via oral route, which increases intestinal load of phosphorus.

The amount of phosphorus contributed by the prescribed medications were generally considered to be a small portion of the recommended daily intake of about 1000 mg/day. Although a few previous studies had reported a serious concern with phosphorus content of medications, they did not report the precise nature of the phosphorus (organic or inorganic) as it may have potential ramifications in intestinal load. Moreover, the previous reports analyzed only the phosphorus content in the medicines prescribed to individual patient cohorts^31,34^.

In order to address this issue, Cupisti et al. conducted a systematic screening of oral medications that are commonly prescribed for patients with CKD. They utilized the Italian medicines agency database to analyze 3763 formulations of 311 distinct APIs and reported that 60 APIs (19.3%) contained at least one phosphorus-containing excipient and a total of 472 formulations (12.5%) contained phosphorus as an excipient. Moreover, it was observed that oral hypoglycemic drug formulations were the most phosphorus-containing drugs (23.8%) followed by antidepressants (19.2%), antihypertensives (17.5%), and gastrointestinal drugs (16.4%). The major finding of the study was that the widely used form of phosphorus in excipient was calcium hydrogen phosphate (77.57%), which has been reported to be exhibiting a lower bioavailability (90–70%) compared with sodium hydrogen phosphate that has a high absorption rate (100%)^36,41^. On the basis of their finding and review of previous reports, they concluded that the phosphorus load in medications may be of concern only in a minor population with distinct medicinal requirements^36^. However, a comprehensive perspective on the potential effects of the additional medicinal phosphorus load in case of the phosphorus absorption and renal clearance by HD has to be considered.

### Absorption and clearance of inorganic phosphorus

Both food additives and pharmaceutical excipients contain inorganic phosphates, which are readily absorbed from the intestine^42–44^, whereas in case of organic phosphates, approximately 60% is absorbed in the intestine^42,45,46^. Although most of the phosphorus-containing food additives such as the sodium tripolyphosphate, sodium pyrophosphate, disodium dihydrogen pyrophosphate, and sodium hexametaphosphate have a 100% bioavailability, the calcium salts used as an excipient have a bioavailability of approximately 80%^41^. Sodium salts of phosphorus are generally used as food quality-modifying, -emulsifying, -dispersing, -buffering, -chelating, -leavening, and -rehydrating agents and others^47^. On the contrary, calcium phosphate and calcium hydrogen phosphate are often used as a filler to combine pharmaceutical ingredients to form a tablet-based solid preparation^43,44,48^.

The amount of phosphorus contributed by medications is minor in most patients; however, in some, it is notable. In patients with CKD, as the renal clearance of phosphorus by HD is limited to approximately 1000 mg/dialysis session, phosphate binders are frequently used to achieve target phosphorus levels. Therefore, even a minor increase in phosphorus intake may have a disproportional effect, which could be seen from two perspectives. From an HD perspective, if a patient is consuming 10 mg of amlodipine of Greenstone brand (phosphorus content: 27 mg), 10 mg of lisinopril from BluePoint laboratories (phosphorus content: 32.6 mg), and 40 mg of Rena-Vite (phosphorus content: 37.7 mg) then the phosphorus load from medication will be 110 mg, which will be 30% higher after considering the dialytic removal, which is a significant phosphorus load for patients with CKD. From the perspective of phosphorus-binding pills, the reported medicinal phosphorus load requires four additional doses of sevelamer or calcium acetate to counter the medicinal phosphorus load.^31,33^ However, this increased pill burden might reduce patient compliance of the prescribed medicines, resulting in lower usage of phosphorus-binder pills, which may have a compounding effect on phosphorus homeostasis^49^. Another important aspect of significance is the phosphorus content of vitamin supplements prescribed for patients with CKD^31^.

The estimated phosphorus load in medications in 90% of the patients with CKD may be <80 mg/day, which is much lower than the phosphorus load from food additives, which could be as high as 800 mg in certain patient populations^34,44^. Nevertheless, medicinal drugs as a hidden source of phosphorus in patients with CKD cannot be neglected.

### Seeking alternatives

If alternative drugs with similar safety, efficacy, and cost exist, they are preferable to be used in place of drugs with high-phosphorus content (such as lisinopril, amlodipine, and bisoprolol) in order to reduce oral phosphorus intake. The probable efficacy of low-phosphorus-containing alternatives could be seen from the perspective of daily phosphorus intake and its removal by dialysis. If a patient undergoing HD has a daily phosphorus intake of 1000 mg, and an HD clearance of about 400 mg/day (1000 mg/dialytic session, thrice weekly), then there will be 600 mg of phosphorus, of which an average of about 60% (360 mg) could be absorbed. If this patient takes 5 mg of amlodipine (Pfizer) and 10 mg of paroxetine (Aurobindo), the absorbable phosphorus will increase by approximately 8.5% (30.5 mg), which is equivalent to 16.1 g of pork (thin)^31^. If both drugs are replaced by alternatives, the intestinal phosphorus load could be decreased by 8.5%, which is a substantial reduction of phosphorus load in patients with CKD. Among the potential alternatives, perindopril, nifedipine, and felodipine have not been reported to contain phosphorus as an excipient^35^. Therefore, perindopril, which is an angiotensin-converting enzyme inhibitor, could be used instead of lisinopril. Similarly, nifedipine and felodipine, which are calcium channel blockers, could be used as potential alternatives to amlodipine^50,51^. Currently, there is a lack of transparency in declaring the excipient phosphorus content by pharmaceutical manufacturers, which slows down the search for alternatives. Therefore, to begin with, all the medications marketed should specify the information regarding the phosphorus content and warnings on its implications on health to both physicians and the dialysis community.

## Lowering dietary phosphorus intake

Although dietary phosphorus consists of organic phosphorus from plant source (e.g., phytates) and animal source (e.g., casein), and inorganic phosphorus (e.g., food additives), only inorganic phosphorus is absorbed from the intestine. The dietary organic phosphorus is converted to inorganic form by intestinal enzymes, which leads to differential absorption of phosphorus from different sources. In case of inorganic phosphorus, 80–100% is rapidly absorbed, whereas only 40–60% of organic phosphorus is absorbed after conversion into inorganic phosphorus^52–54^. Similarly, plant-based organic phosphorus has a low bioavailability (20–40%) as the majority of the phosphorus exists as storage molecule called phytates. Phytates cannot be broken down by humans due to the lack of hydrolyzing enzymes^55^, whereas larger portion of animal-derived phosphorus is present bound to organic molecules such as proteins, phospholipids, and nucleic acids and hence it is easily hydrolyzed and absorbed and has a higher bioavailability (40–60%)^56–58^. Therefore, theoretically, vegetarian diet has a lower phosphate-to-protein ratio leading to decreased phosphorus absorption in comparison with animal-based diet. In a preclinical study, animals fed with grain-based diet had lower serum level of FGF23 and urinary phosphorus excretion when compared with standard synthetic casein-based diet^59^. In a crossover trial of two diets (vegetarian and meat/dairy) in patients with CKD stage late 3 or stage 4, lower serum phosphorus, FGF23 levels, and decreased urinary phosphorus excreted during 24-h excretion was observed in the vegetarian diet compared with the meat-based diet^58^.

In clinical studies, FGF23 was considered as a surrogate marker for serum phosphorus due to its close association in phosphorus homeostasis. The beneficial role of low-phosphorus diet has also been reported from numerous randomized controlled trials (RCTs)^50,51^. Sequestering excess dietary phosphorus using phosphorus binders has also been evaluated as a clinical management strategy. The combined efficacy of low-phosphorus diet and phosphorus binders was evaluated by Isakova et al.^51^ and it was reported that diet combined with phosphate binders was more effective in reducing FGF23 and serum phosphorus compared with single approach. Lowering dietary phosphorus and phosphorus binders were the cornerstone of clinical management. However, poor adherence of patients to phosphorus-restricted diet and hidden sources of phosphorus has reduced the efficacy of this strategy^58^.

###  Diet with low phosphorus–protein ratio

The strategy of maintaining low dietary phosphorus might seem straight forward; however, the major source of dietary phosphorus is proteins, which if restricted, might lead to malnutrition. In addition, it is also a risk factor for death in patients with CKD^25,60^. The estimated phosphorus load from 1 g of protein is around 13–15 mg, out of which 30–70% is absorbed through intestine. Therefore, a 90-g daily intake of protein provides 600–700 mg of phosphorus, which is 70% of the daily recommended intake^40,61^. Therefore, restriction of protein intake being safe both nutritionally and metabolically, it decreases the level of serum phosphorus and slows down the progression of CKD^62–64^. Evidence supporting the benefits of low-protein diet was also provided by a meta-analysis by Fouque et al.^65^, which reported that a low-protein diet containing 0.6–0.8 g protein/kg/day reduces the risk of premature death or kidney replacement by 32%.

In view of the risk of malnutrition and subsequent CKD progression, average protein intake in patients with CKD is measured in terms of normalized protein catabolic rate (nPCR), which is a measure of protein nutritional status^66^. A previous study conducted by Shinaberger et al. reported the best survival rate among patients with normalized protein nitrogen appearance (nPNA or nPCR) between 1.0 and 1.4 g/kg/day whereas greater mortality was observed in nPNA <0.8 or >1.4 g/kg/day^26,67^. Streja et al.^67^ emphasized the interaction between dietary protein intake, PTH levels, and serum phosphorus levels; however, it did not advocate on reducing the nPCR and PTH levels as management strategies for hyperphosphatemia. Furthermore, limiting protein intake is associated with increased risk of death in patients with CKD and MBD as it leads to malnutrition^68,69^.

Therefore, in recent years, studies have recommended the use of phosphorus/protein ratio as an indicator of measuring phosphorus content in food. The concept of phosphorus/protein ratio was proposed by the Kidney Disease Outcomes Quality Initiative (K/DOQI) guidelines for patients with kidney disease to support nutritional counseling and control blood phosphorus levels through diet^3,40^. It is a metric representing ratio of dietary phosphorus (in mg) to protein (in g) for a given food item, which may be more suitable for patients with CKD compared with total protein intake (nPCR). The guideline lists the phosphorus/protein ratios in common food items, which could be used for prescribing healthy diet in patients with CKD. As per the guidelines, dairy products, nuts, beans, and seeds have a high phosphorus–protein ratio and should be avoided. Similarly, meat and tofu have a low phosphorus–protein ratio and are preferable^70^. Guida et al.^71^ used low-phosphorus whey protein concentrate to replace dietary proteins for a short period and observed significant decrease in blood phosphorus and PTH levels, whereas nutrient-related indicators such as albumin did not change significantly. Similarly, Watanabe et al.^24^ found that processed foods contain more protein and phosphate and the higher phosphorus–protein ratio compared with fresh foods. There are various other studies summarizing the protein and phosphorus content and phosphorus–protein ratio of different food stuffs^56,72,73^. In an RCT conducted on patients with ESRD undergoing HD, there was a decrease in serum phosphorus concentration after nutritional counseling on restricting the diet with phosphorus additives and replacing these foods with those with similar nutritional value without the phosphorus additives^74^. Therefore, patients with CKD should be encouraged to consume fresh unprocessed food instead of processed food. In the absence of additive interference, lower the phosphorus–protein ratio, lower the likelihood of hyperphosphatemia. Therefore, patients with CKD who have hyperphosphatemia should choose foods with low phosphorus absorption and low phosphorus–protein ratio^75^.

For educating patients regarding low-phosphorus diet, phosphorus–protein ratio >12 mg/g could be considered as “high-phosphorus food” and <12 mg/g is “low-phosphorus food,” for example, egg white has a phosphorus–protein ratio of only 1.4 mg/g which is classified as a low-phosphorus food, whereas egg yolk has 23 mg/g, which is a high-phosphorus food^56,72^. Some methods followed during cooking, such as soaking foods in water and boiling them helps to reduce the dietary phosphorus content per gram of protein in foods^72^. Therefore, it is recommended that patients with CKD choose foods with a low phosphorus–protein ratio, but with adequate amount of protein to prevent malnutrition, limit the intake of foods high in phosphorous additives, and use of better phosphate binders for the better management of hyperphosphatemia in CKD.

## Summary

Patients with CKD usually consume a variety of oral medications, and these medications may significantly increase phosphorus intake. The lack of transparency by the pharmaceutical manufacturers in providing information on the phosphorus content of the drugs increases the uncertainty of hyperphosphatemia management in patients with CKD. The only way to prevent the risk of phosphorus-containing medications in patients with CKD is by educating doctors and patients on the presence and concentration of phosphorus in the pharmaceutical products. Therefore, pharmaceutical manufacturers should report the phosphorus content/quantity of their products more transparently and in a greater detail. Nephrologists should also take extra caution regarding the phosphorus content of the drugs for patients with CKD to provide evidence-based guidance and management. In addition, the importance of a low phosphorus–protein-ratio diet should be emphasized in dietary education for patients with CKD.
